# Observation on the Effect of Transconjunctival Lower Blepharoplasty Combined With Orbital Fat Release in Middle‐Aged and Elderly Men

**DOI:** 10.1111/jocd.70054

**Published:** 2025-04-30

**Authors:** Chong Lin, Lulu Zhang, Bin Dong, Zhihui Dai, Lei Wu, Li Zhao

**Affiliations:** ^1^ Plastic and Reconstructive Surgery First People's Hospital of Zhengzhou City Zhengzhou China

**Keywords:** lower eyelid pouch, orbital fat release, premaxillary space, tear trough deformity, transconjunctival

## Abstract

**Objective:**

To explore and evaluate the clinical effect of transconjunctival approach combined with orbital septal fat release in correction of lower eyelid pouch in middle‐aged and elderly male patients.

**Methods:**

A retrospective analysis was performed on the clinical data of 17 middle‐aged and elderly male patients with lower eyelid bags underwent this procedure from December 2022 to December 2023. Age ranged from 40 to 60 years old, with an average of 50.6 years old. A transverse incision with a length of 1.0–1.5 cm was made through the conjunctiva, the orbital fat pedicle was dissociated, and the corresponding premaxillary space and prezygomatic space were separated. The pedicled orbital fat flap was placed horizontally in the above space to fill the lacrimal groove. The orbital fat was fixed on the orbital periosteum with absorbable sutures, which were pulled out from the skin and tied for fixation. All conjunctival incisions were not sutured. Postoperative follow‐up was performed, and patient satisfaction was investigated using the visual analog scale to assist in evaluating the clinical effect.

**Results:**

After a mean follow‐up of 5.7 months (range, 3–12 months), the conjunctival incisions healed well in all patients. The lower eyelid bag, tear trough, and palpebral buccal sulcus depression were significantly improved. Satisfaction rate is 94.1% in this study.

**Conclusion:**

The novel technique of using a transconjunctival approach combined with releasing and retaining orbital fat to correct lower eyelid bags in middle‐aged and elderly male patients can achieve good results.

## 1 Introduction

As people age, the lower eyelid becomes enlarged and bloated to varying degrees due to the weakness, relaxation, and loss of tension of supporting structures such as the lower eyelid skin, orbicularis oculi muscle, orbital septum, and canthal ligament. The orbital septum fat bulges and droops in bag‐like shapes, which are called lower eyelid bags, commonly known as eye bags. It can occur in both men and women, but is more common in middle‐aged and elderly people over 40 years old. It is mainly caused by the destruction of the balance between the orbital fat and the lower eyelid support structure, and is often accompanied by aging changes such as sagging of the lower eyelid skin, tear trough deformity, drooping of the midface, and increased crow's feet at the outer canthus [1, 2]. In recent years, the public's demand for beauty has increased significantly. According to the statistics of the International Society of Aesthetic Plastic Surgery (ISAPS) in 2023, among head and facial surgeries, eyelid surgery is the most popular surgery among male beauty seekers, and lower blepharoplasty is a common plastic surgery for middle‐aged and elderly men [3, 4]. With a deeper understanding of eyelid anatomy and advances in medical technology, eyelid surgery techniques are constantly evolving [5]. However, there are not many relevant reports on the research of lower eyelid bags in middle‐aged and elderly men. For middle‐aged and elderly male patients, the traditional transcutaneous lower blepharoplasty may aggravate the patient's tear trough deformity and infraorbital depression by removing loose skin and bulging orbital septum fat. In addition, various complications such as lower eyelid ectropion and blepharon separation often occur after the operation, thus affecting the surgical effect. However, transconjunctival lower blepharoplasty can correct lower eyelid pouches in middle‐aged and elderly patients by removing orbital fat without leaving any scars on the skin. However, this approach has limited effect on the correction of tear trough deformity and cannot improve loose skin and depression in the infraorbital area. At present, the aesthetic trend of lower blepharoplasty focuses on reducing the “eye bags” while reestablishing a smooth transition of the lower eyelid‐cheek interface [6, 7], and improving both periorbital aging and midface aging as much as possible. This requires maintaining or increasing the volume of the eyelid, which can be achieved through fat redistribution or fat transplantation. For middle‐aged and elderly male patients, the main purpose of surgery is to achieve a younger or more aesthetic improvement rather than to correct functional abnormalities, and they have a higher tolerance for skin laxity [8]. Therefore, this study is to explore and evaluate the clinical therapeutic effect of transconjunctival approach combined with orbital septal fat release in correction of lower eyelid pouch. The report is as follows.

## 2 Patients and Methods

This is a retrospective study of the clinical data of 17 middle‐aged and elderly male patients with lower eyelid bags admitted to the Plastic Surgery Department of Zhengzhou First People's Hospital from December 2022 to December 2023. All patients underwent transconjunctival approach lower eyelid bag correction combined with orbital septum fat release. All surgeries are performed by the same surgeon (Z.L.). This study was approved by the Medical Ethics Committee of Zhengzhou First People's Hospital, and all patients signed informed consent. The inclusion criteria for patients in this study were as follows: (1) suffering from varying degrees of orbital fat protrusion in the lower eyelid, accompanied by depression of tear troughs and blepharo‐buccal sulci; (2) having complete clinical data. Exclusion criteria were as follows: (1) suffering from eye diseases; (2) having a history of lower eyelid and/or midface surgery; (3) having previous injections of fillers or fat in the lower eyelid and/or midface; (4) having a history of lower eyelid (eyelid, orbit, or midface) trauma; and (5) having incomplete clinical records. The patients were followed up for 3–12 months after surgery to observe conjunctival congestion, lower eyelid swelling, hematoma, bruising, infection, lower eyelid ectropion, periocular pigmentation, blepharon separation, binocular diplopia, numbness of the lower eyelid, cheek and upper lip, and dry eyes. In addition, postoperative results were evaluated subjectively by patient himself as happy or unhappy with the result by using the visual analog scale (VAS) at the last follow‐up, which was divided into five grades: very satisfied, satisfied, neutral, dissatisfied, and very dissatisfied, with scores ranging from 5 to 1 in turn. Satisfaction rate = (very satisfied + satisfied) cases/total cases × 100%.

### 2.1 Surgical Technique

**FIGURE 1:**
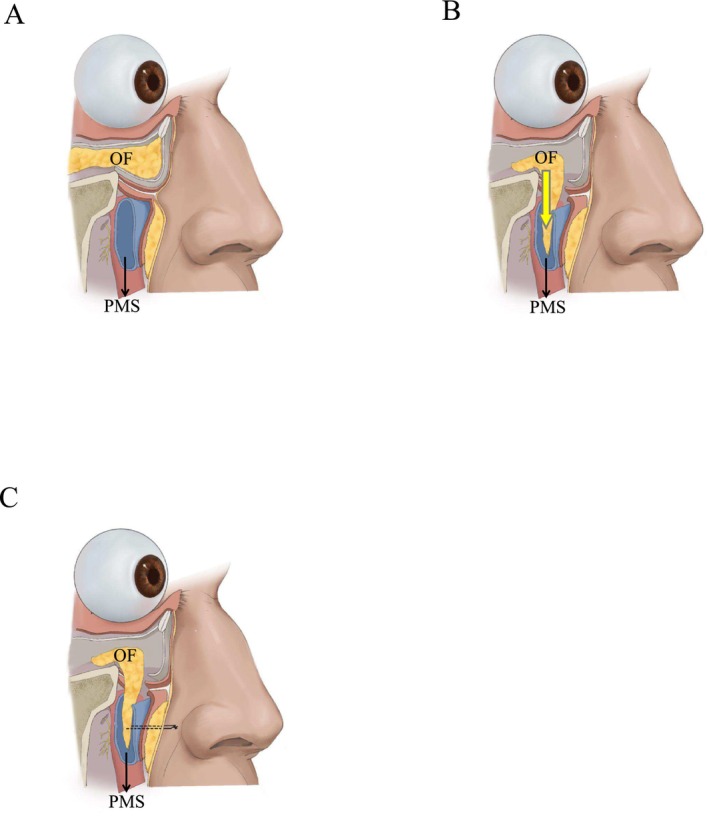
(A) Cross‐section showing orbital fat, retaining ligaments, and the midface soft tissue space. (B) The path of the dissection. Release orbital fat and place it in the facial soft tissue space (PMS). (C) The path of fat fixation. Showing the corrective effect of the tear trough ligament release and fat redistribution after the surgery. (OF, orbital fat; PMS, premaxillary space).

**FIGURE 2:**
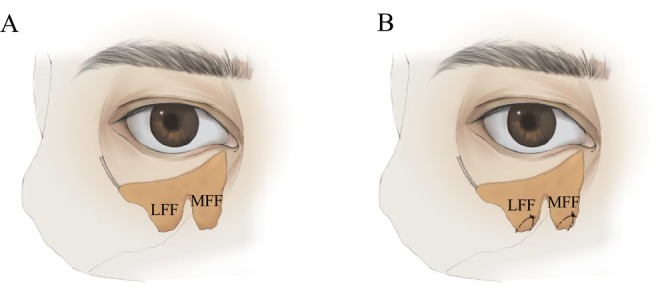
(A) Coronal view: The herniated orbital septum fat was divided into 2 groups: The medial fat flap (medial fat mass only) and the lateral fat flap (including central and lateral fat masses). (B) Coronal view: Place two fat flaps in the facial soft tissue spaces (premaxillary space and prezygomatic space) and fix. (MFF: The medial fat flap; LFF: The lateral fat flap).

**FIGURE 3:**
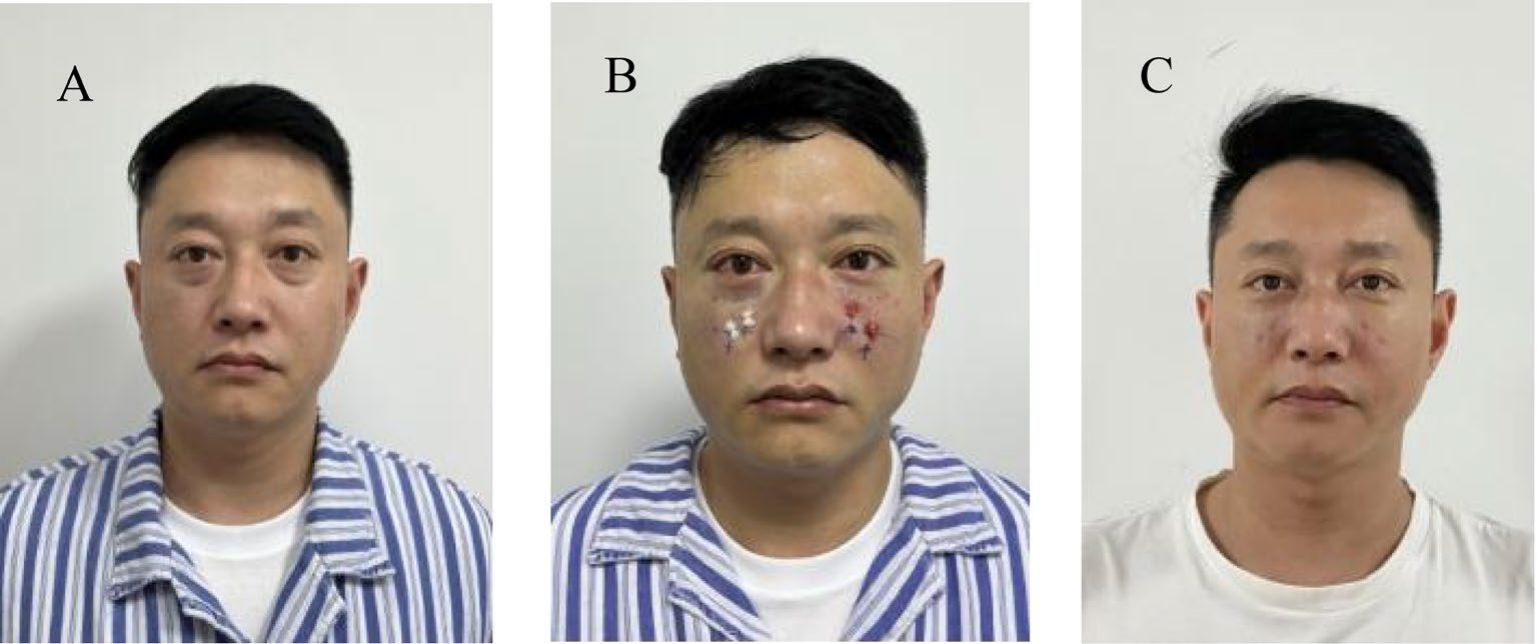
Frontal views of a 40‐year‐old man with lower eyelid bags and tear trough deformity who underwent transconjunctival approach lower eyelid bag correction combined with orbital septum fat release before (A), immediately after (B), and 7 days after (C). Notice the improvement in the lower eyelid contour and tear trough deformity.

**FIGURE 4:**
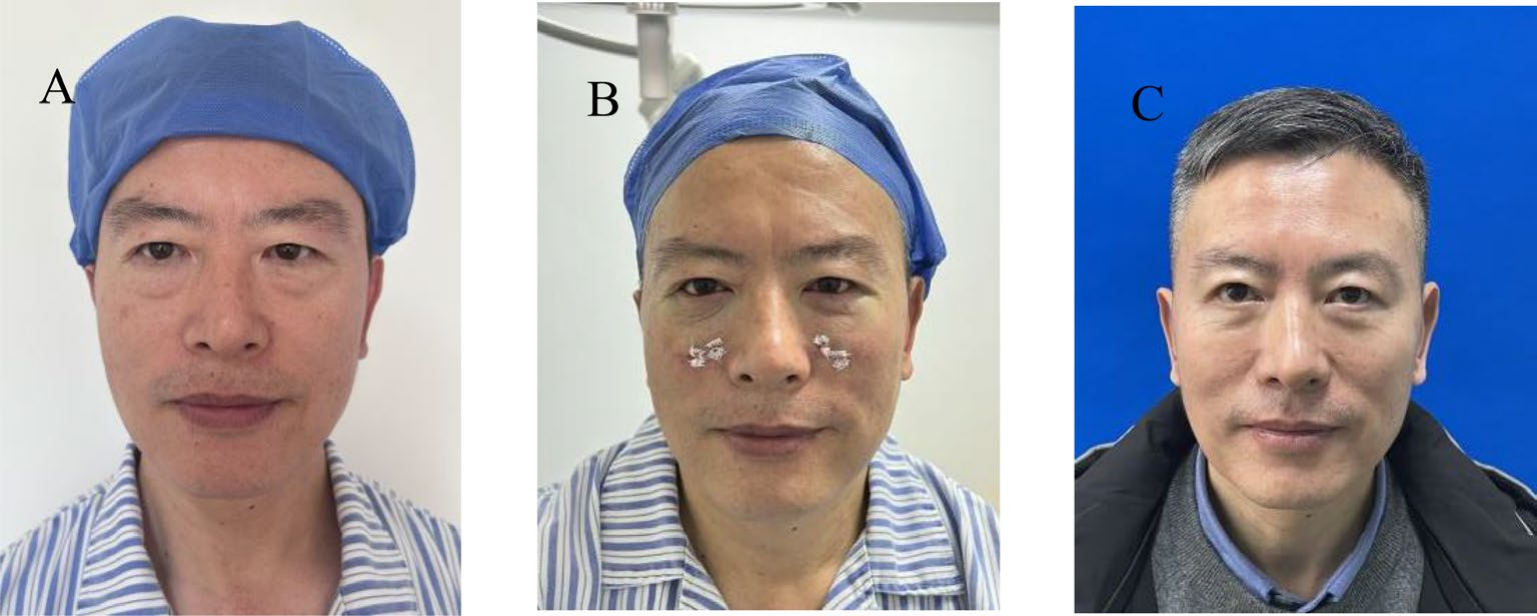
Frontal views of a 50‐year‐old male with lower eyelid bags and tear trough deformity who underwent transconjunctival approach lower eyelid bag correction combined with orbital septum fat release before (A), immediately after (B), and 4 months after (C).

The patient was placed in the supine position, and the protruding area of the orbital septum fat and the infraorbital rim depression were marked. All operations were performed under local infiltration anesthesia. Ask the patient to close his eyes with the eyeball in the upward rotation position. Gently turn the lower eyelid margin over and inject 1–2 mL of 2% lidocaine (containing 1:200000 epinephrine) into the orbicularis oculi muscle and conjunctiva of the lower eyelid fornix. A 1.0–1.5 cm transverse incision was made 5 mm below the tarsal plate, and the orbital septum was exposed by blunt dissection to fully reveal the fat masses. The capsule was completely loosened with a needle electrode to allow the orbital septum fat to fully herniate (Figure 1A,B). The herniated orbital fat mass was divided into two groups of fat flaps by carefully peeling off the surrounding fibrous tissue: the medial fat flap (only the medial fat mass) and the lateral fat flap (including the central fat mass and the lateral fat mass) (Figure 2A). The space between the deep layer of the orbicularis oculi muscle and the superficial layer of the orbital septum was separated downward to the orbital rim to expose the arcuate margin. The tear trough ligament was fully released to enter the premaxillary space. After separating the premaxillary space with the scalpel handle, the scalpel handle was pushed outward to enter the prezygomatic space and fully separate it. The pedicled orbital septum fat flap was placed in the space above to fill the tear trough (Figure 2B). The orbital septum fat was fixed to the orbital periosteum with 6–0 absorbable sutures. The sutures were pulled out from the skin and tied for fixation (Figures 1C, 2B, 3B, and 4B). The conjunctival incision was not sutured.

### 2.2 Postoperative Care

Sterile pressure dressings were applied after surgery, and ice packs or cold compresses were applied to the lower eyelids for 20 min. Antibiotic eye drops were applied for 1 week after surgery, and patients were advised not to rub their eyes or engage in strenuous exercise within 2 weeks. Sutures were routinely removed 7 days after surgery.

## 3 Results

The mean age of the 17 patients was 50.6 years old, ranging from 40 to 60 years old. Postoperative follow‐up was 3 ~ 12 months, with an average of 5.7 months (Table 1). After surgery, the conjunctival incisions of all patients healed well, and lower eyelid bag, tear trough, and blepharo‐buccal sulci depression were significantly improved (Figures 3 and 4). All patients experienced varying degrees of skin petechiae and ecchymoses at the surgical site, which generally subsided gradually over 1–2 weeks and disappeared in about 1 month at the latest. Conjunctival congestion occurred in six patients, who recovered after symptomatic treatment. Two patients experienced mild bleeding in the surgical area 3 days after surgery. Ice compress was applied to stop the bleeding, and the symptoms gradually eased and healed spontaneously. One patient had a sense of induration 3 weeks after surgery, which softened and disappeared 1 to 2 months later. No postoperative complications such as hematoma, infection, diplopia, lower eyelid ectropion, blepharon separation, numbness of the lower eyelid, cheek and upper lip, and dry eyes were observed in any patient (Table 2). One patient had a neutral attitude toward the postoperative effect, and the overall satisfaction rate of this study was 94.1% (Table 3).

**TABLE 1:** Patients characteristics.

Patients(*n* = 17)	
Age (years)	
Mean	50.6
Range	40–60
Gender (male)	17
Diagnosis	
Lower eyelid bags	17
Tear trough deformity	17
Previous operations	0
Follow‐up (months)	
Mean	5.7
Range	3–12
Reoperation	0

**TABLE 2:** Postoperative complications.

Complications	*N* (%)
Conjunctival congestion	6 (35.3)
Lower eyelid swelling	17 (100)
Hematoma	0
Skin petechiae and ecchymoses	17 (100)
Infection	0
Lower eyelid ectropion	0
Periocular pigmentation	0
Blepharon separation	0
Binocular diplopia	0
Numbness of the lower eyelid, cheek and upper lip	0
Dry eyes	0
Induration	1 (5.9)
Bleeding	2 (11.8)

**TABLE 3:** Postoperative satisfaction.

	*N* (%)
Very satisfied	11 (64.7)
Satisfied	5 (29.4)
Neutral	1 (5.9)
Dissatisfied	0
Very dissatisfied	0
Satisfaction rate	94.1%

## 4 Discussion

With the progress of society and the change of aesthetic concepts, the demand for blepharoplasty among men is increasing year by year [4, 9]. Due to the status and role that men hold in society and at work, most of them are reluctant to let others know that they have undergone cosmetic surgery. Moreover, their needs and purposes may be different from those of female patients. Most male patients are more concerned about prominent eye bags, postoperative marks, recovery period, etc., but have a higher tolerance for improvements in sagging skin and wrinkles. They prefer to improve their “tired” appearance through surgery, and on this basis, maintain their man's masculinity at the same time, and will not appear “excessive” plastic surgery. Therefore, the use of conservative methods may be more conducive to keeping male patients maintain a male appearance [8, 10]. Lower blepharoplasty is one of the most common cosmetic surgeries in the world. However, the surgery is challenging because of the complexity of the anatomy, the differences in facial and eyelid aging, the variety of surgical approaches, and the differences in patient expectations. At present, many techniques have been described both at home and abroad, including fat removal, fat transplantation, fat transposition, skin removal, elevation, release of facial ligaments, and so forth, as well as some combinations of surgery, such as lower eyelid bag correction combined with lateral canthus suspension, conjunctival approach lower eyelid bag removal combined with autologous fat injection to fill the tear trough. In addition, some scholars have studied the clinical efficacy of laser treatment or botulinum toxin injection after lower eyelid bag correction surgery [5]. Although lower blepharoplasty has been widely used as a cosmetic procedure, there is no universal consensus on which surgical approach is appropriate for which clinical presentation and which patient. For middle‐aged and elderly men, periorbital aging is not only manifested in lower eyelid bags and sagging lower eyelid skin, but is often accompanied by other manifestations of midface aging, such as tear trough deformity and blepharo‐buccal sulci depression. Through a deep understanding of eyelid and facial anatomy and the principles of aging, the current aesthetic trend is more toward improving periorbital aging while also lifting the midface to a certain extent. Some authors also call it “eyelid lift” [11, 12]. Therefore, for middle‐aged and elderly men, when choosing a surgical approach, periorbital rejuvenation and midface rejuvenation should be achieved simultaneously as much as possible. In addition, fewer complications and a shorter recovery period should also be considered.

In the past, the concept of plastic surgery for periorbital aging focused on the removal of loose skin and orbital fat. Currently, it has shifted to limited release of preserved structures in known facial fat compartments to restore or improve the periorbital and facial “contours” [6]. Therefore, this article aims to achieve periorbital and midface rejuvenation by releasing orbital septum fat to form a pedicled orbital fat flap and utilizing the facial soft tissue space for fat transposition. In recent years, transconjunctival approach has become the first choice for plastic surgeons to treat lower eyelid bags because of its low complication rate and concealed incision [13, 14]. However, transcutaneous blepharoplasty is often plagued by complications such as lower eyelid retraction and lower eyelid ectropion after surgery, which bothers plastic surgeons [15]. In addition, some skin redundancy is acceptable for elderly men, which is usually consistent with their other facial features [8, 10]. Therefore, transconjunctival approach for eyelid bag correction is the preferred procedure in this study.

The concept of fat transposition was first proposed by Loeb, who proposed that fat preservation and fat transposition was more effective than fat removal to achieve a smooth aesthetic transition between the lower eyelid and cheek [16]. In 1998, Goldberg defined a new technique to reposition the medial and central protruding orbital septal fat of the lower eyelid to the subperiosteal space. Advantages of this approach include clear anatomical layers, avoidance of skin incisions and potential intermediate lamellar scarring, and disadvantages include difficult dissection, prolonged edema, and poor long‐term viability of the fat pedicle because of the inadequate vascular supply of the periosteum [17]. Later, Kawamoto and Bradley described a transconjunctival repositioning of fat to the space between the periosteum and the orbicularis oculi muscle to correct lower eyelid bags and tear trough deformity. The advantages of peeling in the superperiosteal plane are that it is relatively easy and fast, and can fully release the tear trough ligament and the orbicularis oculi supporting ligament, but this plane is rich in blood supply, and it is not a true anatomical plane, which may cause accidental damage to important structures, including nerves and blood vessels. Complications may include numbness, intraoperative bleeding, recurrence of eyelid bags, and slow resolution of postoperative ecchymosis and edema [18]. As the understanding of facial anatomy improved, Wong and Mendelson et al. [19, 20] discovered a soft tissue space extending along the horizontal length of the orbital rim and named it the premaxillary space, located below the orbicularis oculi muscle and above the levator labii superioris muscle. They also proposed that free orbital septum fat be transferred to this space to improve periorbital aging. Because this plane is the natural mid‐cheek space, it has the advantages of being loose and easy to peel off, with a wide peeling range, and there are fewer blood vessels and nerves distributed in this plane, resulting in less damage during and after the operation. However, what was used during the operation at that time was free fat, and there was a possibility of fat absorption after the operation, which would affect the long‐term effect. Subsequently, some scholars adopted a transconjunctival approach to release the pedicled orbital septum fat and relocate it in the premaxillary space and prezygomatic space [21]. In addition to the above advantages, this technology can also better retain the periosteum and superficial fat, make it easier to fix the orbital septum fat, and is not prone to retraction after surgery, with good long‐term results. Because it is a natural anatomical space, it is highly safe and can achieve rejuvenation of the periorbital and midface simultaneously. Therefore, in this study, the premaxillary space and prezygomatic space were used as the dissection plane for fat transposition in the transconjunctival approach for lower eyelid bag correction. In addition, we also made a brief summary of the differences in the above three different planes (Table 4).

**TABLE 4:** Comparison of fat transposition to different planes.

	Advantages	Disadvantages
Subperiosteal plane	Clear anatomical layers; avoid skin incisions.	Difficult dissection; prolonged edema; inadequate vascular supply.
Superperiosteal plane	Peeling easy and fast; fully release ligaments; adequate vascular supply.	Damage to important structures; multiple complications.
PMS and PZS	Natural mid‐cheek space; peeling easy and with a wide peeling range; safe: less damage during and after the operation; achieve rejuvenation of the periorbital and midface.	Long operation time; high technical requirements for surgeons.

Abbreviations: PMS, premaxillary space; PZS, prezygomatic space.

In this study, the conjunctival incisions of all patients healed well. By comparing the preoperative and postoperative photos of the patients, the eyelid bags, tear trough deformity, and palpebral buccal sulcus depressions were significantly improved (Figures 3 and 4). There were no lower eyelid ectropion, orbicularis oculi paralysis, blepharon separation, diplopia, or numbness of the cheek or upper lip after the operation. Lower eyelid ecchymosis and swelling were the most common postoperative complications and resolved in up to 4 weeks. The skin of the lower eyelid of individual patients was relatively loose and wrinkles increased after surgery, but the patients said that it was acceptable. We also suggested that the patients should undergo radiofrequency therapy in the later stage to reduce the degree of skin relaxation.

Finally, we all know that lower blepharoplasty is a common surgery, but with the refinement of anatomy and innovation of concepts, we must also keep pace with the times, explore the clinical application of different surgical methods, discover the problems therein and continuously improve and optimize them. This study proposed the use of a transconjunctival approach combined with the transposition of orbital septum fat to correct lower eyelid bags for middle‐aged and elderly men, and achieved relatively satisfactory results. However, there are still some limitations in this study. The sample size included in this study is relatively small, which has a certain impact on the generalizability of the research results. Future studies can include larger and more diverse populations. In addition, this study is not a comparative study, and we can conduct comparative studies on the efficacy with other surgical methods in the future.

## 5 Conclusions

For middle‐aged and elderly male patients, the transconjunctival approach is used to release and retain orbital septum fat, and the facial soft tissue gap is used for fat translocation to correct lower eyelid bags, improve tear troughs, and reconstruct eyelid‐cheek fusion. This provides surgeons with a safe, effective, and long‐lasting surgical method that can simultaneously achieve periorbital and midface rejuvenation and minimize the occurrence of complications.

## Author Contributions

C.L., L. Zhang, and Z.D. collected data. C.L. analyzed data and wrote the manuscript. L. Zhoa designed the research study and performed the research. B.D. and L.W. provided the conceptual guidance. All the authors reviewed and commented on the manuscript and agreed to its contents.

## Ethics Statement

This study was approved by the Medical Ethics Committee of Zhengzhou First People's Hospital.

## Conflicts of Interest

The authors declare no conflicts of interest.

## Data Availability

The data that support the findings of this study are available on request from the corresponding author. The data are not publicly available due to privacy or ethical restrictions.
